# Imaging Cherenkov emission for quality assurance of high-dose-rate brachytherapy

**DOI:** 10.1038/s41598-020-60519-z

**Published:** 2020-02-27

**Authors:** Katsunori Yogo, Akihiro Matsushita, Yuya Tatsuno, Takahiro Shimo, Seiko Hirota, Marika Nozawa, Shuichi Ozawa, Hiromichi Ishiyama, Hiroshi Yasuda, Yasushi Nagata, Kazushige Hayakawa

**Affiliations:** 1 Graduate School of Medicine, Nagoya University, 1-1-20 Daiko-minami, Higashi-ku, Nagoya, Aichi 461-8673 Japan; 2 Graduate School of Medical Science, Kitasato University, 1-15-1 Kitasato, Minami, Sagamihara, Kanagawa 252-0373 Japan; 3 Department of Radiology, Tokyo Nishi Tokushukai Hospital, 3-1-1 Matsubara-cho, Akishima, Tokyo, 196-0003 Japan; 4 Department of Radiation Biophysics, Research Institute for Radiation Biology and Medicine, Hiroshima University, Kasumi 1-2-3, Minami-ku, Hiroshima, 734-8553 Japan; 5 School of Medicine, Kitasato University, 1-15-1 Kitasato, Minami, Sagamihara, Kanagawa 252-0373 Japan; 6 Hiroshima High Precision Radiotherapy Cancer Center, 3-2-2 Futabanosato, Higashi-ku, Hiroshima, 732-0057 Japan; 7 Department of Radiation Oncology, Hiroshima University Hospital, 1-2-3 Kasumi, Minami-ku, Hiroshima, 734-8551 Japan

**Keywords:** Optics and photonics, Applied physics, Quantum physics, Techniques and instrumentation

## Abstract

With advances in high-dose-rate (HDR) brachytherapy, the importance of quality assurance (QA) is increasing to ensure safe delivery of the treatment by measuring dose distribution and positioning the source with much closer intervals for highly active sources. However, conventional QA is time-consuming, involving the use of several different measurement tools. Here, we developed simple QA method for HDR brachytherapy based on the imaging of Cherenkov emission and evaluated its performance. Light emission from pure water irradiated by an ^192^Ir γ-ray source was captured using a charge-coupled device camera. Monte Carlo calculations showed that the observed light was primarily Cherenkov emissions produced by Compton-scattered electrons from the γ-rays. The uncorrected Cherenkov light distribution, which was 5% on average except near the source (within 7 mm from the centre), agreed with the dose distribution calculated using the treatment planning system. The accuracy was attributed to isotropic radiation and short-range Compton electrons. The source positional interval, as measured from the light images, was comparable to the expected intervals, yielding spatial resolution similar to that permitted by conventional film measurements. The method should be highly suitable for quick and easy QA investigations of HDR brachytherapy as it allows simultaneous measurements of dose distribution, source strength, and source position using a single image.

## Introduction

High-dose-rate (HDR) brachytherapy is a superior cancer treatment method that delivers a higher dose with a steep dose distribution to tumours in a shorter treatment time thus requiring fewer fractions, compared with external beam radiation therapy^1^. In HDR brachytherapy, a highly active source, typically an ^192^Ir γ-ray source, is placed directly in the patient’s prescribed region of treatment (within or near the tumour). Using a wire inside an implanted flexible catheter tube, or solid tube (applicator), the Ir source is placed for treatment using an HDR treatment machine. The treatment machine can deliver the source remotely using computer-controlled methods during the treatment time (remote-afterloading system). The dose distribution for HDR brachytherapy is delivered by controlling the source position and dwell time. A typical cervical cancer HDR brachytherapy treatment consists of three applicators, typically moving the source to ~5 positions with an interval of ~10 mm with a typical dwell time per position of ~20 s.

However, even small errors can cause severe complications in normal tissues because of the high source activity^2,3^. Errors in source position and dwell time can cause errors in the prescribed dose^4^. Quality assurance (QA) with respect to source movement is thus essential for ensuring the safety of HDR brachytherapy^5–7^.

In the last decade, image-guided brachytherapy (IGBT) has become widely used to improve clinical outcomes^8–11^. In addition, the development of dose optimisation methods utilising planning based on patient anatomy and with optimised source movements has allowed for dose delivery and dose distributions customised for individual targets in patients^12–16^. The importance of QA is increasing with these advances in HDR brachytherapy as it tends toward smaller steps and significantly shorter dwell times for highly active sources, which are driven using remote afterloaders^17,18^.

In radiation therapy, treatment plans are planned with a treatment planning system (TPS), which calculates dose distribution in the patient body, and the plans are converted to treatment parameters (such as source positions and dwell times for brachytherapy), and then transferred to a treatment machine^1^. The goal of quality assurance (QA) for radiation therapy is to ensure that each treatment is consistently administrated and that the radiation oncologist’s clinical intent, i.e., planned dose distribution, is accurately realised while guaranteeing patient safety^5–7^. The actual dose administered by a given treatment plan is checked using phantoms, such as a water tank, which are surrogates of the patient body with simple and controllable geometry, prior to the treatments. Correct workings of the treatment machine are also regularly checked to ensure that the parameters are within tolerance^5–7^.

Conventional QA for HDR brachytherapy is performed using a combination of several tools^5–7^. For example, source strength measurements are commonly performed using a well-type ionisation chamber, with standard protocols recommended in the literature^19,20^. Regarding source position measurements, methods with the autoradiograph (film) and a specified ruler are recommended as standard methods in the literature^6,7,21^.

In addition, several QA tools have been developed and proposed. The source parameters of dwell position and dwell time can be measured using different tools and apparatuses. These include X-ray fluoroscope systems^22^, diamond detectors^23^, flat panel detectors^24^, plastic scintillators^25^, films and photodiodes^26^, diode arrays^27^, and video cameras^28^.

The development of QA tools for HDR brachytherapy has focused on measuring the dwell time and dwell position of the source for tracking. The American Association of Physicists in Medicine (AAPM) TG-56 report recommends that, in addition to source position and timing, the dose distribution should be measured accurately^6^. Comparing the dose calculated by the treatment planning system (TPS) with the results of measurements performed before treatment is necessary to reduce errors.

In this respect, Manikandan *et al*. used commercial ion chamber arrays to measure the dose distribution^29^. However, in their study, the spatial resolution was limited to the 0.76-cm pitch of the detectors. Further, while Smith *et al*. used an electronic portal imaging device for brachytherapy source dosimetry, this involved several corrections, such as those related to the energy, source-to-detector distance, and incident angle^30^. Espinoza *et al*. developed a two-dimensional (2D) diode array to measure the 2D dose map and perform pretreatment QA for HDR brachytherapy^31^. 2D dose maps of 10 × 10 cm^2^ dose maps with a 0.5 × 0.5 mm^2^ pixel size could be calculated using the in-house software based on the dwell positions and times as measured with the diodes, which had a detector pitch of 10 mm.

Although several QA tools and techniques have been developed, direct measurements of the dose distribution are still conventionally performed using films, such as radiochromic films^32–34^. GAFchromic^™^ films, which exhibit tissue equivalence and high spatial resolution, have been employed for the QA of HDR brachytherapy; however, their use involves developing time. Films also must be loaded and unloaded for each exposure. In addition, they require a nonlinear calibration curve to convert the optical density into the dose. These QA procedures are labour-intensive and time-consuming.

At present, no available technique can quickly and readily provide information regarding the dose distribution as well as the source position and source strength in real-time using stand-alone tools specialised for HDR brachytherapy. The development of a QA tool providing real-time images of the dose distribution at specific times would allow rapid feedback, thus permitting error correction during the QA process itself. For the quick and precise measurement of dose during HDR brachytherapy with high spatial resolution, monitoring of the Cherenkov emission is particularly interesting. For a charged particle (such as an electron) traveling in a medium at a velocity exceeding that of light in the medium, Cherenkov radiation is emitted along the trajectory of the particle. Several previous works have reported the use of Cherenkov emission as a QA tool in photon and electron beam radiation therapy^35–39^. Cherenkov emission has potential utility in visualising the dose distribution in water via visible light imaging without using fluorescent dyes. However, dose measurements for photon and electron beam with directional beams in previous works have required corrections.

**Figure 1:**
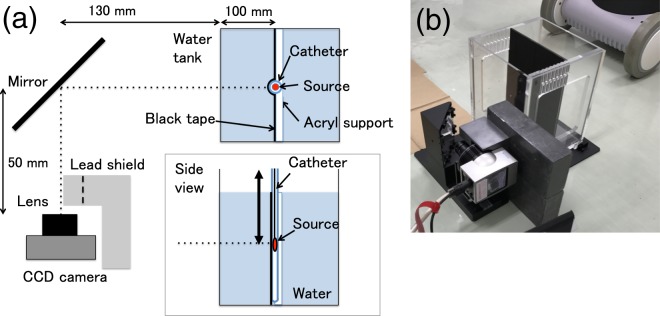
(**a**) Schematic (**a**) and photograph (**b**) of experimental setup. The dashed line on the lead shield shows the aperture of the lead block.

In this study, we developed a QA method based on imaging Cherenkov radiation generated in water irradiated with an ^192^Ir source, consisting of a water tank and a CCD camera (see Fig. 1). We propose the use of water itself, a safe-to-use reference material for radiation therapy, as a detector that can convert the invisible radiation dose to visible light emission for fast QA during HDR brachytherapy. This method allows measurement of the dose distribution, source strength, and source position simultaneously from a single image during HDR brachytherapy QA^5–7^. We produced a method and evaluated its performance for light intensity, such as dose dependence, repeatability, and dose rate dependence, to confirm the feasibility of using the method for HDR brachytherapy QA. Further, the Cherenkov emission was also simulated using Monte Carlo (MC) calculations.

We evaluated the Cherenkov profiles, comparing with dose distributions calculated from the treatment planning system (TPS). Source strength was measured as the intensity of Cherenkov light as a function of the time after the source installation to the facility and half time was calculated from the decay curve. The source positions, as measured based on the light captured from water, were compared with those measured by the conventional film and ruler method. In addition, we characterised the spatial resolution, minimum detectable dose, and corresponding dwell time of the method.

## Methods

### Method for imaging Cherenkov emission

The Cherenkov emission imaging tool consists of a water phantom and a CCD camera (Fig. 1(a)). A 200 mm × 200 mm × 250 mm water phantom was prepared from a 5-mm-thick piece of transparent poly(methyl methacrylate) (PMMA). Deionised pure water supplied by a purification system (Elix Advantage, Merck KGaA, Darmstadt, Germany) was used to fill the phantom in order to eliminate light emissions arising from possible contaminants in the water. A custom-made PMMA holder with dimensions of 200 mm × 200 mm × 10 mm and 2-mm-diameter grooves for fixing the catheter was placed in the water phantom parallel to the movement axis of the source using a holder. The holder and catheter were covered with black tape (T743-2.0, High-Performance Masking Tape, Thorlabs, Inc., NJ, USA) unless otherwise noted. All measurements were taken at the middle groove.

The Cherenkov light produced by the irradiation of the device with an ^192^Ir source was reflected by a mirror and recorded with a cooled CCD camera (BU-50LN, BITRAN CORPORATION, Gyoda, Japan)^40^. The camera was placed perpendicular to the source axis and set 35 cm away from the source centre. The spatial resolution of the obtained images was 0.17 mm/pixels. The camera was Peltier-cooled to 0 °C, and the imaging rate was 17 fps. A lens (LM12JC1M, Kowa Optical Products Co., Ltd., Tokyo, Japan) with focal length f = 12 mm, fixed at F = 3 was used, where F is the f-number of the optical system, defined as the ratio of the lens’ focal length to the diameter of the entrance pupil. The distance of the image was calibrated by recording a metric ruler under light room conditions, placed immediately in front of the holder in the water tank fully filled with water. To test the distortion of the system, the pattern of ticks was evaluated in pixel units. The extent of distortion in the optical system, evaluated as the difference between the actual distance and predicted distance of the tick at the end of the image, was less than 1 pixel (0.17 mm). The camera was controlled remotely from outside the treatment room using a personal computer connected through a cable. The recorded digital images were saved in the tagged image file format (TIFF) as 16-bit greyscale images with a size of 772 × 580 pixels. The camera was shielded with 10-mm-thick lead blocks to reduce scattering radiation incident on the camera. The setup was covered with a box and a black curtain to eliminate background light.

Before capturing the light images, background images were recorded without irradiation during the same cumulative time using the same setup for capturing the light images. The background images were subtracted from the light images and processed using a median filter with a 2-pixel radius. Light intensity profiles along and away from the central axis of the source movement were analysed. The light intensity profiles were obtained using the software package ImageJ with 1 pixel in both directions.

The light intensity profiles, given as grey values, were converted into absolute dose profiles by using calibration factors (slope of the dose linearity, i.e., light intensity/dose) at r = 10 mm, where r is defined as the distance away from the source centre axis. The profiles were calibrated to r = 10 mm, because this position is reference point, where dose is prescribed during cancer treatment, according to the AAPM guideline^19,20^. The 10 mm point is a typical and representative position of the cancer target. Hence, attention was mainly paid to the dose at this point. Note that the dose along and near the source axis (r = 0) is included inside of the source and the applicator tube (typically r = 1–5 mm); these doses are clinically not considered because these regions do not include the cancer.

The dose linearity at r = 10 mm was investigated in terms of the light intensity at the point, corresponding to the reference point dose in the range of 0.3–20.0 Gy, which corresponds to accumulated doses over positions of 4.3–289.4 Gy. An accumulated dose is approximately 14 times larger than a point dose. The slope of the dose linearity was analysed by fitting into a linear function using the least squares method with OriginPro software (OriginLab Corp., MA, USA). The light profiles are also normalised at r = 10 mm, comparing them as relative values.

Source strengths are measured as light intensity at the midpoint of the full width at half maximum (FWHM) of the source position in light intensity profiles^25^.

### Dose calculations and Monte Carlo simulations

Simulations of the Cherenkov emission and the amount of energy deposited in water (dose) were performed using GEANT4^41^, and the obtained distributions were compared with the measured ones. The dose calculations in this study were confirmed to be consistent with those previously reported within a range of uncertainty^42–44^.

The light distributions (calibrated into dose profiles) were compared with the dose distributions calculated by the TPS (Oncentra v4.3, Nucletron). 2D dose matrices with 1-mm slice thicknesses were exported and accumulated to the thickness of 100 mm because the observed light imaged with the camera was the light projected in a 100-mm-thick water layer in front of the source.

### Experiments

An ^192^Ir source (Nucletron mHDR- v2, γ-rays; mean energy of 0.355 MeV; half-life of 73.83 days; dimensions of 4.5 mm × 0.9 mm) was transported in the catheter comprising a flexible plastic tube (Lumencath, 6 F Applicator, Nucletron) to the water tank. The source position and dwell time were controlled using an HDR brachytherapy unit (microSelectron HDR-V3, Nucletron). The delivered dose was controlled by varying the dwell time, which was calculated according to the dose calculation protocol in TG-43^19,20^. The reference source strength was measured using a well-type chamber according the standard method^19,20^. The source strengths varied during all experiments from air-kerma strength = 35,300 down to 15,600 cGy cm^2^ h^−1^ (U), which corresponds to 320–140 GBq.

Light images during irradiation were obtained when the ^192^Ir source was stopped at the image centre. For the Cherenkov imaging, a dose of 6 Gy was delivered at the reference point, located 10 mm away from the source centre as defined in TG-43^19,20^, unless otherwise noted. The typical source dwell time was 58 s; the cumulative camera time was 78 s, using a 32,800-U ^192^IR source. The dose rate at the reference point was ~6 Gy/min. The measurements were performed within a few hours on the same day, unless otherwise noted. All light from the water and the catheter was recorded without covering the catheter. The light from the catheter itself was also measured with the uncovered catheter placed in air (i.e. water tank without water).

For the dose linearity and repeatability measurement, doses were varied between 0.3 and 20 Gy (reference point dose). For the dose rate dependence measurements, the same dose of 10 Gy was delivered with varied dwell times (91–206 s) and different dose rates (on different days). Presented data were normalised to the average intensity of all measurements. For the decay curve measurements, the source strength as light intensity was measured using the same dwell time (240 s) and the same cumulative time (260 s) on different days. For the source position measurements, the source was set to stop at 1434 mm away from the treatment machine surface delivering the source. For the positional interval measurements, the source was stopped at two points on either side of the image centre at different intervals of 2.5 and 40 mm to measure the source intervals. The typical cumulative time at a single position was 150 s, using a 31,000 U ^192^IR source. The source positions were determined as the midpoint of the FWHM. The source interval distance was taken as the distance between source points 1 and 2. The source positions and intervals measured from the light images were compared with those measured with two standard methods, the film and ruler methods^6,7^. We used two pieces of a radiochromic film (RTQA2, Ashland Inc., Wayne, NJ, USA) to measure points 1 and 2 independently. The ruler specific to the treatment machine was supplied by the vendor (Nucletron). All measurements of source positions were repeated for four times and averaged. Spatial resolutions of the source position measurements are defined as the standard deviation of these measurements.

## Results and Discussion

### Imaging of Cherenkov emission

**Figure 2:**
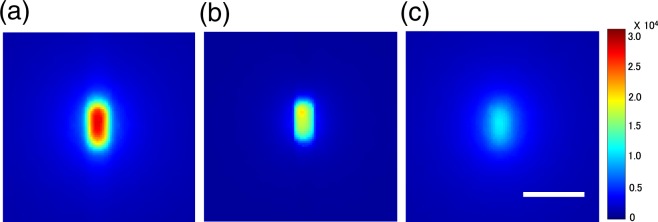
Images of light emission from water irradiated with ^192^Ir source. (**a**) Image of light from pure water and catheter irradiated with source. (**b**) Image of light from the catheter in air when irradiated with source. (**c**) Image of light from pure water irradiated with source after the catheter was covered with tape. Scale bar: 5 mm. Exposure time was 58 s. Images are expressed in grey values in 16-bit scales as in colour bars.

An image of all light emission produced by irradiation with the Ir source transported in a catheter placed in pure water is shown in Fig. 2(a). This includes the light emitted from the catheter itself in addition to that from the water, as confirmed by an image of the light emission produced by irradiating the catheter with the source in air (i.e. absence of water) as shown in Fig. 2(b). Figure 2(c) depicts the light emitted from the pure water after the catheter was covered with black tape and then irradiated with the Ir source. Thus, this image presumably represents the light emitted from the pure water alone.

**Figure 3:**
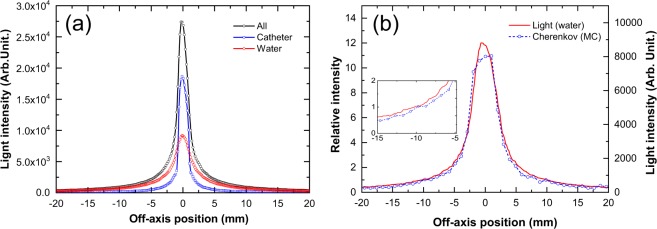
Representative profiles of light emissions from water irradiated with ^192^Ir source. (**a**) Light from pure water irradiated with source after catheter was covered with black tape (water). Data are compared with those from light from uncovered catheter in water (all) and light from catheter in air when irradiated with source (catheter). (**b**) Measured light emission data compared with those from Monte Carlo (MC) calculations. Data are normalised relative to those at the position = 10 mm. The data are zoomed in the inset.

Figure 3(a) shows the lateral light intensity profiles of the light emitted from the water alone (covered catheter in water) along with the profiles measured from all emission (uncovered catheter in water) and the catheter alone (uncovered catheter in air), derived from the images in Fig. 2. The light intensity of all emission integrated over all positions is approximately three times higher than that of water alone, while the light intensity of the catheter is approximately two times higher than that of water alone.

The addition of the light intensity of water and that of the catheter yielded almost the same result as that of all emission (difference within 2%), supporting the idea that the light recorded under the shading of the catheter is composed of light from water alone. Our interest is on the dose in water.

The result that the observed light from the catheter is nearly twice as intense as that from water suggests the detector’s sensitivity to light depends on the emitting material’s refractive index. The material’s refractive index is directly related to the phase velocity of light therein and therefore the minimum energy of electron capable of yielding Cherenkov light. The thresholds for yielding Cherenkov light are 146.3 keV for plastic and 264.1 keV for water^35^.

The γ-photons from the Ir source have a mean energy of 355 keV, which is close to the Cherenkov light emission thresholds. This is unlike previous studies on Cherenkov emission imaging, where emissions were produced by radiation with energy on the megaelectronvolt scale^35–39^. The γ-photons emitted from the Ir source have energies either greater or lesser than the Cherenkov threshold, depending on the material being irradiated. For the subsequent experiments, we covered the catheter, which is outside of the cancer region, to measure the dose distribution in water related only to the Cherenkov emission from water.

Figure 3(b) shows the measured light intensity profiles normalised to dose profiles at the position r = 10 mm, as well as Cherenkov light profiles obtained from MC calculations. The MC curve near the peak skewed presumably due to statistical uncertainty of the calculations (~10%) and/or the asymmetry arising from the wire attached to the source. The differences between the measured profiles (water alone) and that based on the MC calculations are within 30%. These data suggest that the light from the water irradiated by the Ir source can primarily be attributed to the Cherenkov emission.

**Figure 4:**
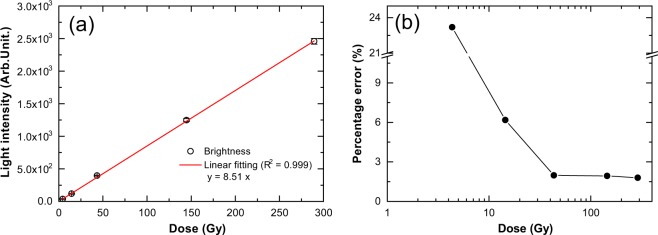
Characteristics of measured light intensity of water. (**a**) Light intensity as a function of dose. (**b**) Percentage error of intensity as a function of dose. Percentage errors are calculated relative to the averaged values for four repeated measurements, i.e., (standard deviation)/(averaged value). Dose presented are accumulated considering water thickness, *t* = 100 mm. Water thickness is the perpendicular distance between the source and the water tank wall near the camera.

We characterised light intensity performance to use the Cherenkov method for the QA of brachytherapy. Figure 4(a) shows the measured light intensity response as a function of the dose at a reference point located 10 mm from the source centre^19,20^. The light intensity was linearly proportional to the source dwell time, i.e. to the dose delivered at the reference point (0.3–20.0 Gy), and accumulated doses (4.3–289.4 Gy), with R^2^ > 0.99. We also evaluated the repeatability of the light intensity (percentage difference in measured light intensity) as a function of the accumulated dose (see Fig. 4(b)). The error bar in the figure represents the standard deviation (s. d.) of four repeated measurements.

The repeatability of the light intensity remains within 2% for reference doses of more than 3 Gy (reference point dose), which corresponds to accumulated doses of 43.4 Gy. Thus, the dose of 3 Gy satisfied the requirement of detecting dose differences within 3.0%. For dose distribution measurements, no obvious threshold has been set in brachytherapy. Nevertheless, we adopt a typical ~3.0% dose difference according to the literature on photon beam therapy^33^. This dose corresponds to the typical dwell time of 27 s for source strengths of 35,300 cGy cm^2^ h^−1^ (U).

### Comparison of Cherenkov profiles and dose distributions

**Figure 5:**
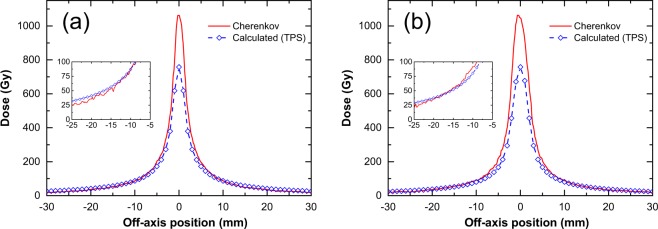
Comparison of light intensity profiles calibrated to dose with dose distributions calculated using TPS along directions (**a**) perpendicular and (**b**) parallel to source movement. Calibration to dose are performed by a fit of Cherenkov vs dose at r = 10 mm as in Fig. 4(a). Dose presented are accumulated considering water thickness, *t* = 100 mm. Water thickness is the perpendicular distance between the source and the water tank wall near the camera. The data are zoomed in the insets.

Figure 5 shows the light intensity profiles of the Cherenkov emission, calibrated to absolute dose at r = 10 mm, observed in water alone and compares them with the dose distributions calculated using the TPS. The profiles shown are obtained parallel and perpendicular to the movement of the source (source axis). The dose profile in the perpendicular direction is sharper than that in the parallel direction, reflecting the fact that the cylindrical source has a larger height than diameter.

The dose profiles measured with Cherenkov light generally agree with the calculated dose distributions in both directions, except for small distances in the region around the source centre (see Fig. 5). The agreements of Cherenkov dose profiles are, on average, within 5% and are less than 10%, for 7 mm < r < 30 mm. However, for a dose less than ~7 mm from the source centre, the discrepancy rapidly exceeds 10%. These differences are larger than the typical ~3.0% dose difference criterion for photon beam therapy^33^, although no obvious criterion for patient plan dose has been set for brachytherapy. TPS saturation at the maximum at the centre (r = 0) is due to the upper limitation of dose calculation in the setting of the TPS to avoid higher doses, which might cause radiation injuries to the patient, and to save calculation time.

In addition, the TPS dose is affected by the limitation of dose calculation accuracy at short distances near and inside the source^6,44^. The TPS calculations and the MC data installed in the TPS were verified to represent the actual dose within ~3% measured with several methods for a point 2.5 mm away from the source centre^6,44^.

Several studies on Cherenkov emissions produced by therapeutic radiation beams have been performed, especially for external beams with energies in the megaelectronvolt range^35–39^. While these Cherenkov emissions were useful for visualising the radiation dose, they did not agree with the dose distributions. The difference between Cherenkov emission profiles and TPS calculations are within ~13–20% for 6 MV x-ray photon beams^35,45^ and within ~60% for 6 MeV electron beams^39^. To collect these data, the following measurement conditions were used: the depth range was 0–10 cm for photons and 0–5 cm for electrons, and the angle of view was approximately 25 degrees for photons and approximately 15 degrees for electrons. Previous works focused on improving these discrepancies and resolving issues arising from imaging of anisotropic Cherenkov light, which originate from the directionality of MV beams. These methods included using corrections^35^, converting the anisotropic Cherenkov light into isotropic fluorescence^45,46^, and improvement of the optical system (conventional lens to telecentric lens)^47^.

The Cherenkov emission distribution in the water irradiated with the Ir source is more similar to the TPS dose distributions than those reported in previous studies on Cherenkov emission imaging of external radiotherapy beams^35–39^ (Fig. 5). One of the merits of using this method in brachytherapy is that the dose distribution of the Cherenkov emission agrees with the dose distribution using conventional lens, without requiring any corrections or addition of a fluorophore. This indicates that the method has potential utility for evaluating the distribution of the dose delivered by HDR brachytherapy units. The method can also compare delivered doses to those determined by TPS calculations without additional corrections, indicating high suitability for the QA of patient plans.

The similarity in between the Cherenkov emission distributions and TPS dose distributions can be attributed to properties of isotropic radiation from the brachytherapy source. γ-rays from the 192Ir source, through the process of radioactive decay, are emitted randomly and spread into all directions (isotropic rays). Thus, γ-rays from the source essentially do not possess anisotropic properties to be considered for the imaging of these lights, except for the asymmetry originating from the source capsule structure. Furthermore, we do not need an anisotropy correction, because the angle variation over our field of view (+/−3 cm) was very small^35^.

**Figure 6:**
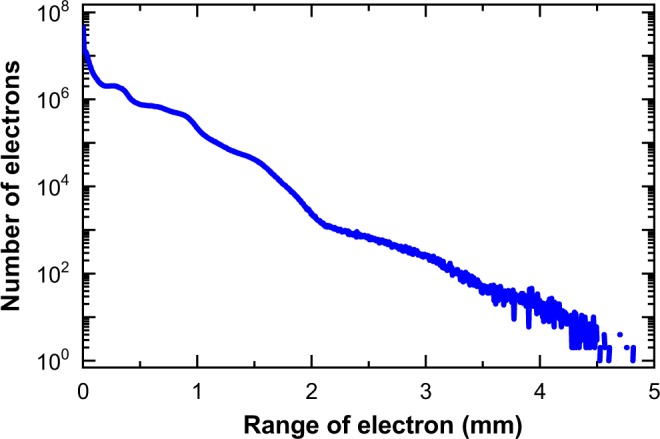
Histogram of range of electrons produced by γ-rays and of β-rays from Ir source. Calculations were performed using Monte Carlo methods (GEANT4).

Not, only γ-photons, but also electrons (beta particles) are directly emitted from 192Ir source upon decay. The encapsulation of the 192Ir source has almost sufficient thickness to stop these electrons, stopping 99.97% of electrons. However, residual electrons escape the capsule and contribute to the dose (approximately 0.05% compared to gamma-ray dose). These electrons can be observed in Fig. 6 as electrons with a long range, on the order of millimetres. Bremsstrahlung photons generated by these beta electrons also reach the water and generate electrons above the Cherenkov threshold. These source electrons partially explain the difference between the Cherenkov (red) and calculated (TPS) (blue) profiles, especially for r < 2.5 mm in Fig. 5 ^44^.

### Source strength and position measurements

**Figure 7:**
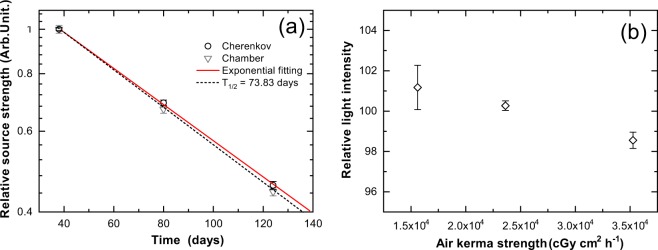
Source strength measured as light intensity (**a**) Decay curve of measured Cherenkov light intensity. A decay curve of ^192^Ir (half-life T_1/2_ = 73.83 days) is presented for comparison. The reference source strength was measured using a well-type chamber (chamber). (**b**) Relationship between light intensity and measured dose rate of brachytherapy source. Error bars represent standard deviations for four repeated readings. All measurements are normalised with respect to averaged intensity over all data (n = 12).

Figure 7(a) shows the source strength measured as Cherenkov light intensities as a function of the number of days after the installation of the source. The source strength decreased with time after source installation, exhibiting a typical exponential decay (radiation decay) curve. The Cherenkov intensity decay curve was fitted using an exponential function that yields a half-decay time of 76.8 ± 1.3 days, ~4% larger than the half-life of 73.820 ± 0.009 days^48^. We also compared the Cherenkov data with data from a well-type ionization chamber.

For measuring source strength at different days, we should measure the Cherenkov light intensity with different dose rates. Thus, we evaluated the dose rate dependency of the Cherenkov light intensity, and the results are shown in Fig. 7(b). It can be seen that the light intensity is not highly dependent on the dose rate, exhibiting a deviation of less than 3% for dose rates of 15,600–35,300 cGy cm^2^ h^−1^ (U). This satisfies the requirement that the source strength should be measured within 3%^6^.

To check source position accuracy, at least, a single source position should be compared with the expected positions^6^. We measured the source position using the Cherenkov light images and compared them with the expected position. We also compared the results with those of standard methods using the film and the specified ruler, which is recommended in the literature^6,7^. The deviation of the source position measured with the Cherenkov method from the expected position was within 0.2 mm, and it was comparable with the deviation within 0.5 mm measured for the film and 0.2 mm for the ruler. The spatial resolutions are 0.3 mm for the Cherenkov method, 0.1 mm for the film, and 0.2 mm for the ruler.

**Figure 8:**
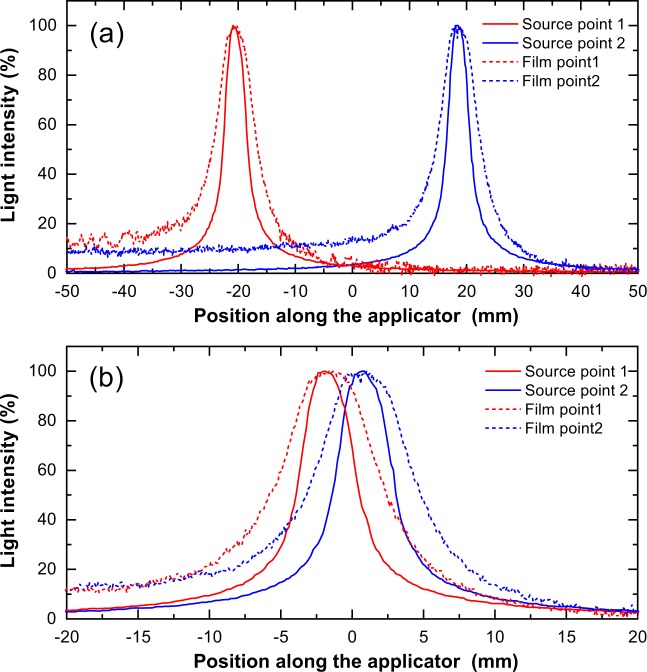
Source position measurements. Profiles of light from pure water compared to those from film measurements: (**a**) preset interval = 40 mm and (**b**) preset interval = 2.5 mm. Note that the range of position is expanded in (**b**) for clarification.

In addition, we measured the source position intervals using the Cherenkov light images and compared them with those obtained using conventional film and ruler measurements. Figure 8 shows the light intensity profiles of the Cherenkov emission obtained while stopping the source at two points for different preset intervals and compares them with the profiles measured using film. The interval between the source positions as measured from the Cherenkov light images is 40.2 ± 0.5 mm (mean ± s. d.) for a preset interval of 40 mm (Fig. 8(a)). These values are comparable to those obtained from the film (39.8 ± 0.4 mm) and ruler (40.4 ± 0.2 mm). Further, for a preset interval of 2.5 mm, the source interval as measured from the light images was 2.5 ± 0.1 mm. These values are comparable to those obtained from the film (2.3 ± 0.4 mm) and ruler measurements (2.6 ± 0.2 mm).

## Conclusion

The results of performance testing of the method confirmed the feasibility of using Cherenkov emission for the QA of HDR brachytherapy in the recommended manner^5–7^. The developed method has several advantages over conventional QA methods. Our method allows the simultaneous measurement of dose distribution, source strength, and source position using a single image. In conventional QA, these parameters are measured separately using different tools in a time-consuming manner. The method allows efficient repeated measurements using the same configuration at different source positions and/or for different source dwell times, unlike films that require loading and unloading for each exposure to the source. By enabling dose visualisation at specific times, the system provides rapid feedback for error correction during the QA process.

One of the concerns of using a CCD camera with a radiation field is the change in the response of the CCD element over time with radiation exposure. We have used this tool for over 3 years (exposure time of approximately 10 hours and total doses of approximately 90,000 Gy into the water tank), and the CCD element did not show severe damages. Light linearity responses per dose did not change during the 180 days period within 2%. However, effects from larger doses with HDR brachytherapy should be investigated. We propose two QA processes to ensure that the performance of this set-up does not change over time. One is a quick check of the existence of ‘dead’ or ‘broken’ elements (pixel(s)), which would occur as dark spots under bright conditions when uniformly illuminated, or white spots under dark conditions in the same place permanently. The other is confirming that dose linearity slopes (light intensity response against dose) do not change within 2%.

While we believe that the method is highly suited for the QA of HDR brachytherapy, we note that some issues still require resolution. We found that the method allows measurement of source dwell positions with similar precision as that of conventional methods based on Cherenkov emission imaging. However, the resolution of the measurements of the source dwell time is limited to the exposure time of ~60 s. The real-time, simultaneous, and accurate confirmation of the dwell positions and dwell times of the radiation source would be very useful for the QA of HDR brachytherapy. However, improvements in the time resolution of the system are necessary to determine the source movement in real time (i.e. the video rate). Such improvements can be achieved by capturing brighter images using a camera with an image intensifier or an electron-multiplying charge-coupled device (CCD).
